# Obesity has a detrimental impact on temporal decline of sperm quality in normospermic patients: insights from a cross-sectional study of 2,430 patients over 14 years

**DOI:** 10.1186/s12958-025-01495-9

**Published:** 2025-12-22

**Authors:** Giorgio Ivan Russo, Maria Giovanna Asmundo, Andrea Cocci, Ali Saber Abdelhameed, Annalisa Liprino, Filippo Giacone, Debora Lombardo, Antonino Guglielmino, Sandrine Chamayou

**Affiliations:** 1 Urology Section, University of Catania, Catania, Italy; 2 Urology Section, University of Florence, Florence, Italy; 3 Department of Pharmaceutical Chemistry, College of Pharmacy, King Saud University, P.O. Box 2457, Riyadh, 11451 Saudi Arabia; 4 Unità di Medicina della Riproduzione, Centro HERA, Catania, Italy

**Keywords:** Sperm, Infertility, Age, Male, Semen analysis, Obesity

## Abstract

**Background:**

Obesity is a known factor influencing sperm quality through hormonal imbalances and oxidative stress, contributing to male infertility.This study examines the association between obesity and temporal declines in sperm quality in normospermic men over 14 years.

**Methods:**

We conducted a retrospective analysis of 2,430 normospermic men who underwent fertility evaluation between 2010 and 2024. BMI categories were defined per WHO criteria, and semen analysis was performed following WHO guidelines.

**Results:**

The study found that higher BMI correlated with declines in sperm quality. In normal-weight men, significant decreases were observed in total sperm count (*r*= -0.0020, *p* = 0.004), motility AB (*r*= -0.0396, *p* < 0.001), volume (*r*= -0.2007, *p* = 0.001), and morphology (*r*= -0.0210, *p* = 0.005). Overweight men showed reductions in sperm motility (A + B) (*r*= -0.0246, *p* = 0.009) and volume (*r*= -0.1616, *p* = 0.015), while obese men exhibited notable declines in TSC (*r*= -0.0029, *p* = 0.010) and volume (*r*= -0.2238, *p* = 0.009). Overall, obesity was associated with progressive declines in key sperm parameters over time.

**Conclusions:**

Obesity negatively impacts sperm quality in normospermic men, with significant declines in TSC, motility, and morphology. These findings underscore the importance of maintaining a healthy BMI to support male reproductive health and suggest the need for interventions targeting obesity-related fertility issues.

## Introduction

Obesity has a well-documented impact on sperm quality and male fertility, influencing various physiological and hormonal pathways that are essential for reproductive health [1]. Excess body fat contributes to hormonal imbalances, including reduced testosterone levels and increased estrogen, both of which are known to impair spermatogenesis and reduce sperm production [2]. Studies have shown that the endocrine profile in obese men often includes higher levels of estradiol, as adipose tissue facilitates the conversion of testosterone to estrogen, leading to a feedback suppression of the hypothalamic-pituitary-gonadal axis and further reducing testosterone levels [3].

In addition, obesity is closely linked to increased oxidative stress and chronic low-grade inflammation, both of which can have detrimental effects on sperm quality. Elevated oxidative stress can lead to sperm DNA fragmentation, an important factor in male infertility, as DNA integrity is crucial for fertilization and embryo development [4]. The inflammatory state associated with obesity has also been shown to influence sperm morphology and reduce motility, which are vital parameters for successful fertilization [4–6].

While numerous epidemiological studies suggest a link between obesity and impaired male fertility, some research contradicts the negative effects of BMI on semen quality.

Hyperinsulinemia, hyperleptinemia, chronic inflammation, and oxidative stress are important mediators of obesity that may affect the male reproductive system.

Obesity is a well-established factor impairing male fertility and reproductive function, primarily through disruptions of the hypothalamic-pituitary-gonadal (HPG) axis, impaired testicular steroidogenesis, and metabolic imbalances involving insulin, cytokines, and adipokines. Specifically, obesity negatively influences key semen parameters—such as sperm concentration, motility, viability, and morphology—through these pathophysiological mechanisms [7]. Moreover, it contributes to increased sperm apoptosis, DNA fragmentation, altered chromatin condensation, and epigenetic modifications, some of which may be transmitted to the offspring [7].

Obesity has emerged as a significant contributor to male infertility, not only through systemic endocrine disruption but also via multiple local and molecular mechanisms that impair spermatogenesis and sperm function. One of the key pathways involves the increased expression of cytochrome P450 aromatase in adipose tissue, which enhances the peripheral conversion of androgens to estrogens [8]. This hormonal shift results in negative feedback on the hypothalamic-pituitary-gonadal (HPG) axis, leading to reduced gonadotropin-releasing hormone (GnRH), luteinizing hormone (LH), and follicle-stimulating hormone (FSH) secretion, ultimately compromising intratesticular testosterone production and Sertoli cell support for spermatogenesis.

In addition to endocrine disruption, obesity-induced scrotal adiposity impairs thermoregulation, promoting testicular hyperthermia. Elevated testicular temperature has been shown to disrupt the blood-testis barrier, alter germ cell apoptosis, and impair meiotic division, contributing to reduced sperm quality. Moreover, obesity is associated with a chronic pro-inflammatory state and increased production of reactive oxygen species (ROS), which generate oxidative stress within the testicular microenvironment [9]. This oxidative burden results in sperm DNA damage, including the formation of mutagenic DNA adducts and the accumulation of 8-hydroxy-2’-deoxyguanosine (8-OHdG) [10]. The DNA repair enzyme OGG1 may excise these lesions, generating abasic (AP) sites; however, the concomitant downregulation of AP endonuclease activity in obesity impairs proper repair, rendering these sites susceptible to strand breaks, especially under additional stressors such as heat [10].

Beyond structural DNA damage, oxidative stress may also induce epigenetic alterations in spermatozoa, including aberrant DNA methylation and histone modifications [11]. These changes may not only compromise fertilization potential and early embryo development but also have transgenerational effects on offspring health. Collectively, these interlinked pathophysiological mechanisms highlight the complex and multifactorial impact of obesity on male reproductive health, extending well beyond mere hormonal imbalance.

In this context, a study examined 411 men undergoing IVF, grouped by BMI, and found no significant differences in semen analysis parameters between groups. However, overweight and obese men had significantly fewer good-quality embryos compared to normal-weight and underweight men, although other IVF outcomes, including total embryo count and clinical pregnancy rates, were unaffected by BMI [12].

Male obesity negatively impacts fertility by reducing sperm quality [13], increasing difficulties in natural conception, and potentially worsening assisted reproductive technology outcomes, although findings regarding sperm retrieval success remain inconsistent. Weight loss may reverse some adverse effects on male fertility by improving semen parameters and reproductive hormone levels. Additionally, paternal obesity is associated with gestational complications, offspring developmental abnormalities, and epigenetic alterations that may extend adverse health effects into subsequent generations [14].

Moreover, the accumulation of fat negatively affects spermatogenesis. Studies indicated that patients with low phase angle, had detrimental sperm parameters in particular lower sperm concentration and total sperm count [6]. Since electricity flows more easily through hydrated tissues like muscle, appropriate PA values are likely to be indicators of better body composition [15]. Together, these physiological and environmental factors lead to reduced sperm count, poorer sperm morphology, and diminished overall reproductive health, potentially resulting in decreased fertility and challenges with conception [16].

While the impact of obesity on male fertility is well established, the specific effects of obesity on sperm quality in normospermic men remain insufficiently explored. This gap in research is significant, as understanding how obesity influences sperm parameters in men who do not exhibit overt sperm abnormalities may provide insights into subclinical reproductive issues. Therefore, the objective of this study is to evaluate the potential detrimental impact of obesity on sperm quality in normospermic men, addressing an important, yet understudied, aspect of male reproductive health.

## Materials and methods

We conducted a retrospective analysis of semen analysis results obtained at a single institution as part of an infertility evaluation from 2010 to 2024. The study included patients aged 18 years and older seeking fertility assessment. A total of 2,430 normospermic patients have been included in this analysis. We gathered data on each patient’s age and performed a physical examination, recording measurements for height, weight, and BMI. The study protocol was approved by the Institutional Review Board at Centro HERA - UMR (Approval No. 1/2023), and all participants provided informed consent at enrollment.

We conducted a retrospective analysis of semen analysis results obtained at a single academic institution between January 2010 and March 2024. The study population included all male patients aged 18 years and older who underwent semen analysis as part of an infertility evaluation. No additional inclusion or exclusion criteria were applied. This unselected approach was intended to capture a real-world population reflective of the general cohort of men presenting for fertility assessment.

Semen analysis has been conducted based on 5th and 6th WHO edition. Semen samples were collected by masturbation into a sterile container after a sexual abstinence period of 2–7 days. Analyses were conducted immediately after liquefaction and included evaluations of seminal volume, sperm concentration, progressive motility, and morphology.

Body Mass Index (BMI) has been classified according to WHO criteria into categories such as underweight (BMI < 18.5), normal weight (BMI 18.5–24.9), overweight (BMI 25-29.9), and obesity (BMI ≥ 30).

Oligozoospermia was defined as < 39 million or < 15 million spermatozoa per ml; asthenospermia was defined as motility A + B < 32%, and teratozoospermia was defined as normal morphology < 4%. Oligoasthenoteratozoospermia (OAT) was defined by the presence of all three abnormalities [17, 18].

To evaluate the association between BMI and semen parameters (e.g., volume, concentration, motility, morphology), we performed linear regression analyses. Each semen parameter was modeled as a continuous dependent variable, with BMI entered as the primary independent variable. Model assumptions, including linearity, homoscedasticity, and normality of residuals, were verified using standard diagnostic plots. All statistical analyses were conducted using Stata (Stata Statistical Software: College Station, TX: Stata Corp LP), with statistical significance set at *p* < 0.05. Normally distributed continuous variables were reported as median (interquartile range, IQR), and group differences were assessed using Student’s t-test or the Mann–Whitney U-test, depending on distribution (assessed by the Kolmogorov–Smirnov test).

## Results

Table 1 summarizes baseline characteristics of a cohort, with median and interquartile range (IQR) values for key variables. Participants had a median age of 44 years and a BMI of 26.1 kg/m². Sperm parameters include a median total sperm count of 131.3 million, concentration of 48 million/ml. Additional metrics include pH (median 8) and morphology (11% normal forms)​ (Table 1).

**Table 1:** Baseline characteristics of the cohort

Variable	
Age (years), median (IQR)	44 (40, 49)
BMI (Kg/m^2^), median (IQR)	26.1 (24.1, 28.9)
Total sperm count (million), median (IQR)	131.3 (84, 202.9)
Sperm concentration (million/ml), median (IQR)	48 (32.5, 76)
Leucocytes, median (IQR)	0.1 (0, 0.42)
Volume (ml), median (IQR)	2.8 (2, 3.7)
Motility AB (%), median (IQR)	50 (45, 58)
Motility C (%), median (IQR)	16 (11, 21)
Motility D (%), median (IQR)	32 (26, 39)
pH, median (IQR)	8 (7.9, 8.2)
Morphology (%), median (IQR)	11 (7, 27)

*IQR* interquartile range, *BMI* body mass index

In the whole cohort, 877 (36.09%) had normal weight, 1,126 had overweight (46.34%) and 427 (17.57%) had obesity.

The Table 2 reveals several significant findings when comparing normal weight, overweight, and obese groups. In terms of reproductive parameters, total sperm count decreases from 135.9 million in the normal weight group to 125 million in the obese group, though this change is not statistically significant (*p* = 0.10). Sperm concentration, however, does show a significant reduction across groups, with values of 50 million/ml in the normal weight group and 45.6 million/ml in the obese group (*p* < 0.01). Other parameters, including leucocyte count, semen volume, motility (AB, C, D types), pH, and morphology, do not differ significantly between groups, suggesting BMI has limited impact on these specific aspects of sperm quality.

**Table 2:** Clinical and sperm parameters according to BMI classification

Variable	Normal weight (BMI < 25) *N* = 877	Overweight (BMI ≥ 25 and < 30) *N* = 1,126	Obese (BMI ≥ 30) *N* = 427	*p*-value*
Age (years), median (IQR)	43 (40, 48)	44 (40, 48)	45 (40, 50)	0.02
BMI (Kg/m^2^), median (IQR)	23.5 (22.2, 24.2)	27 (25.9, 28.4)	32 (30.6, 34.6)	< 0.01
Total sperm count (million), median (IQR)	135.9 (88, 210)	132.99 (85.99, 207.74)	125 (78.12, 192.57)	0.10
Sperm concentration (million/ml), median (IQR)	50 (34.65, 79.2)	48 (32, 75)	45.6 (31, 70)	< 0.01
Leucocytes, median (IQR)	0.1 (0, 0.42)	0.1 (0, 0.42)	0.1 (0, 0.5)	0.96
Volume (ml), median (IQR)	2.8 (2, 3.7)	2.9 (2, 3.8)	3 (2, 3.8)	0.30
Motility AB (%), median (IQR)	50 (45, 58)	50 (45, 58)	51 (45, 58)	0.95
Motility C (%), median (IQR)	17 (11, 23)	16 (10, 21)	17 (11, 21)	0.39
Motility D (%), median (IQR)	32 (26, 39)	32 (26, 39)	32 (26, 38)	0.60
pH, median (IQR)	8 (7.9, 8.2)	8 (7.9, 8.2)	8 (7.9, 8.1)	0.97
Morphology (%), median (IQR)	11 (6, 30)	12 (7, 30)	10 (6, 26)	0.16

*IQR* interquartile range, *BMI* body mass index

*Kruskall-Wallis test

When performing regression analysis between sperm parameters and years of sample collection, in individuals with normal weight (BMI < 25), we observed significant negative associations with total sperm count (*r*= -0.0020, *p* = 0.004), motility AB (*r*= -0.0396, *p* < 0.001), volume (*r*= -0.2007, *p* = 0.001), and morphology (*r*= -0.0210, *p* = 0.005). However, sperm concentration does not show a significant effect (*p* = 0.288).

For overweight individuals (BMI ≥ 25 and < 30), sperm motility (A + B) (*r*= -0.0246, *p* = 0.009) and volume (*r*= -0.1616, *p* = 0.015) are negatively affected, though total sperm count, concentration, and morphology show no significant effects.

**Fig. 1:**
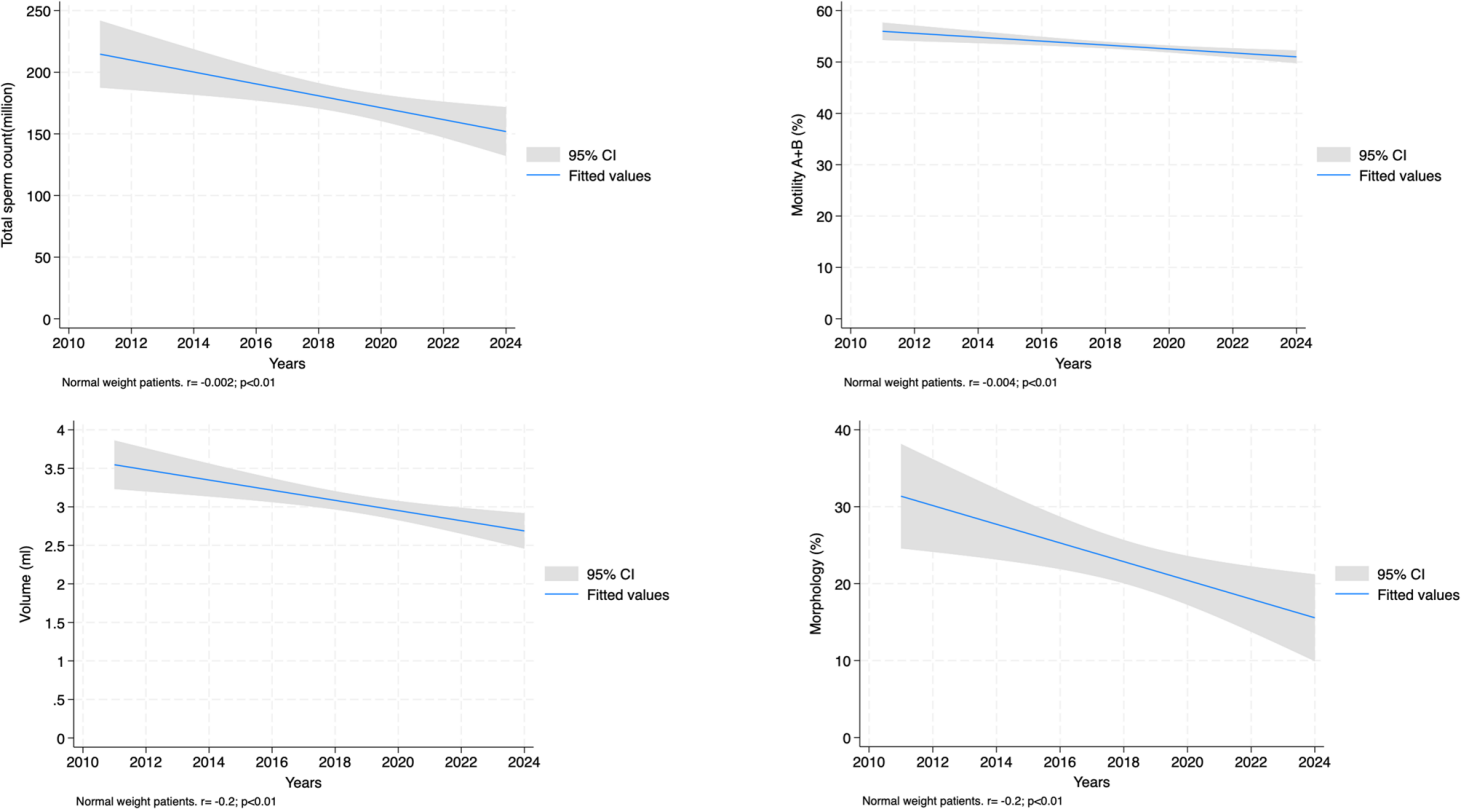
Regression analysis between years and sperm parameters in normal weight patients

**Fig. 2:**
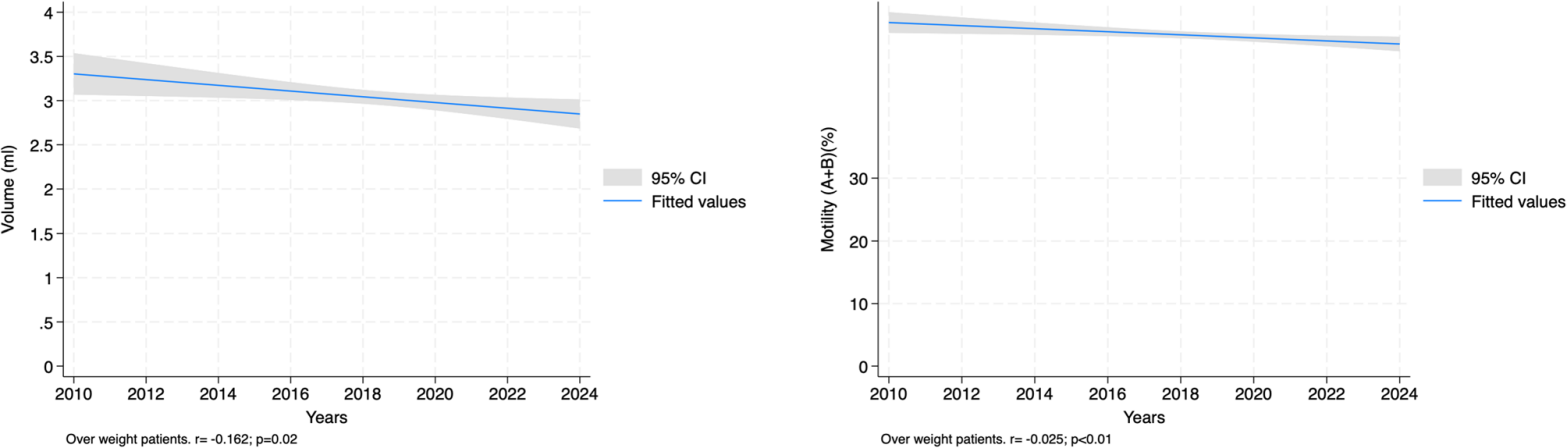
Regression analysis between years and sperm parameters in over weight patients

**Fig. 3:**
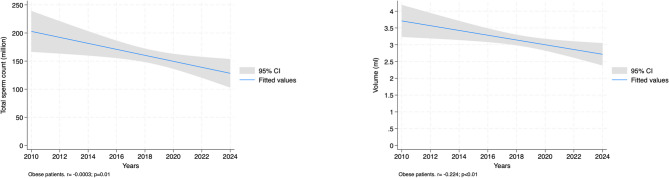
Regression analysis between years and sperm parameters in obese patients

In the obese group (BMI ≥ 30), there is a significant negative association with total sperm count (*r*= -0.0029, *p* = 0.010) and volume (*r*= -0.2238, *p* = 0.009), but no significant effect on sperm concentration, motility AB, or morphology (Table 3 and Figs. 1, 2, 3.

**Table 3:** Temporal trend analysis of sperm parameters using regression analysis according to BMI classification

BMI Group	Variable	Coefficient	95% CI	*p*-value
Normal weight (BMI < 25) *N* = 877	Total sperm count (million)	-0.002	[-0.003, -0.001]	< 0.01
	Sperm concentration (million/ml)	-0.002	[-0.006, 0.002]	0.29
	Motility AB (%)	-0.040	[-0.061, -0.018]	< 0.01
	Volume (ml)	-0.201	[-0.316, -0.086]	< 0.01
	Morphology (%)	-0.021	[-0.036, -0.006]	< 0.01
Overweight (BMI ≥ 25 and < 30) *N* = 1,126	Total sperm count (million)	-0.001	[-0.002, 0.001]	0.30
	Sperm concentration (million/ml)	-0.001	[-0.004, 0.003]	0.78
	Motility AB (%)	-0.025	[-0.043, -0.006]	< 0.01
	Volume (ml)	-0.162	[-0.292, -0.031]	0.02
	Morphology (%)	-0.011	[-0.025, 0.004]	0.12
Obese (BMI ≥ 30) *N* = 427	Total sperm count (million)	-0.003	[-0.005, -0.001]	0.01
	Sperm concentration (million/ml)	-0.002	[-0.008, 0.005]	0.63
	Motility AB (%)	0.007	[-0.024, 0.038]	0.65
	Volume (ml)	-0.224	[-0.392, -0.056]	< 0.01
	Morphology (%)	-0.009	[-0.034, 0.017]	0.51

*IQR* interquartile range, *BMI* body mass index

Figures 1, 2 and 3 demonstrate that the steepest declines in semen quality over the study period were observed in individuals with normal weight, whereas overweight and obese individuals showed a plateau or delayed onset of decline, suggesting potentially distinct pathophysiological dynamics in different adiposity strata.

## Discussion

The influence of body mass index (BMI) on male fertility is a topic of increasing scientific interest due to its potential implications for reproductive health. Obesity, in particular, has been shown to adversely affect sperm quality, and studies have highlighted its role in disrupting endocrine, metabolic, and inflammatory pathways essential for normal spermatogenesis.

Levine et al. demonstrated a significant decline in sperm counts (as measured by sperm concentrations and total sperm count) between 1973 and 2011, driven by a 50–60% decline among men unselected by fertility from North America, Europe, Australia and New Zealand [19].

Our study sought to examine the impact of BMI on sperm parameters in normospermic men, specifically focusing on total sperm count (TSC), sperm concentration, motility, morphology, and volume, while exploring the potential impact of obesity on these parameters. In fact, these findings are consistent with these secular trends: in our cohort of normozoospermic men from 2010 to 2024, we observed a statistically significant decline in total sperm count, motility, and morphology over time, particularly in the normal-weight subgroup.

Our study findings suggest a significant negative impact of BMI on sperm quality across different categories of BMI. These findings align with previous research that has consistently demonstrated that higher BMI correlates with declines in male reproductive health, particularly in terms of sperm motility, morphology, and sperm count. However, the exact mechanisms by which BMI influences sperm parameters remain complex and multifactorial, involving both hormonal imbalances and physiological alterations that are characteristic of higher body fat levels.

The present study reports that men in the normal weight category (BMI < 25) exhibited notable declines in sperm count, motility, volume, and morphology, despite having a generally healthy BMI. In contrast, overweight and obese men (BMI ≥ 25) showed more consistent reductions in total sperm count and volume, suggesting that BMI may influence different sperm parameters at varying levels of severity. The significant associations between increased BMI and impaired sperm quality suggest that deviations from normal weight could contribute to fertility problems, potentially indicating subclinical reproductive dysfunction.

Kasman et al. conducted a systematic review calculating a model to evaluate the association between temporal trends in obesity/temperature and sperm count. Authors found that from 1973 to 2011, obesity contributed an estimated 1.8% to the overall decline in sperm counts. When adjusted to reflect the highest reported obesity impact from any study, this contribution rose to 7.2%. Regionally, obesity-related declines in total sperm counts were most pronounced in the USA at 9.9%, followed by Europe at 3.1%, Asia at 1.9%, while New Zealand showed a slight decrease of -0.4%. When analyzed by fertility status, BMI contributed 1.7% to declining sperm counts in men with unknown fertility status and 2.1% in men seeking fertility evaluation [20].

While our study similarly observes a temporal decline, our stratification by BMI allows for additional granularity. Notably, we found that BMI influences the slope of sperm decline differently across parameters. For example, sperm motility (grades A + B) and volume were significantly affected in overweight men, while total sperm count and volume were most affected in obese men. These differences suggest that the relationship between obesity and male fertility is multifaceted and may evolve over time.

These findings underscore the variation in obesity’s impact on sperm counts based on geographic region and fertility status, highlighting the roles of regional health factors and lifestyle influences on male reproductive health.

A key factor in the relationship between obesity and sperm quality is the disruption of hormonal balance. As adiposity increases, so does the conversion of testosterone into estrogen, a process mediated by aromatase, an enzyme found in adipose tissue [21]. This hormonal imbalance results in lower levels of testosterone, which plays a critical role in spermatogenesis. Previous studies have demonstrated that reduced testosterone levels, along with elevated estrogen, can lead to a suppression of the hypothalamic-pituitary-gonadal axis, impairing the production of sperm in the testes [2, 3]. The lower testosterone levels not only hinder spermatogenesis but may also contribute to the reduced sperm motility and morphological abnormalities observed in obese individuals.

The present study’s findings are consistent with this concept, as we observed significant negative associations between BMI and sperm count and morphology in both normal weight and obese categories. These findings suggest that the hormonal effects of obesity, even in the absence of overt clinical symptoms, may have subtle but significant impacts on sperm quality. For instance, the significant decline in sperm morphology in overweight and obese patients could be attributed to the hormonal imbalances affecting sperm development.

Numerous studies have reported a decline in semen quality, particularly sperm concentration (SC) and total sperm count (TSC). A previous meta-analysis showed significant declines in SC and TSC among men from North America-Europe-Australia (NEA), but data from South/Central America-Asia-Africa (SAA) were insufficient at that time. The current study conducted an updated global meta-analysis, combining data from 223 studies (1973–2018), revealing substantial decreases in SC and TSC worldwide, with an accelerated decline after the year 2000. Specifically, the SC among unselected men globally dropped by 51.6% between 1973 and 2018, and the annual rate of decline more than doubled post-2000. These findings emphasize the need for further research into potential causes and clinical implications of the observed global decrease in sperm quality [22].

Obesity adversely impacts fertility and correlates with various obesity-related markers and sperm parameters in men This study found significant differences among body mass index groups: mean waist circumference, body fat mass, and waist-to-height ratio were notably higher in obese individuals, with a negative relationship between sperm motility and waist-to-height ratio in obese males (*r* = -0.447).

Beyond hormonal dysregulation, obesity is also associated with increased oxidative stress and chronic low-grade inflammation. Both of these factors are known to negatively impact sperm quality. Oxidative stress, resulting from an imbalance between reactive oxygen species (ROS) and antioxidant defenses, has been implicated in sperm DNA fragmentation, which compromises sperm function and fertilization capacity [23].

Limitations of this study include. The retrospective nature of this study means data was collected and analyzed after the events had occurred. This approach can introduce biases, as retrospective analyses rely on previously recorded data that may lack important information or consistency. Consequently, some confounding variables might not have been adequately controlled, which could influence the study’s conclusions about the relationship between BMI and sperm quality. Although the study evaluated key sperm parameters (sperm count, motility, morphology, volume), other crucial factors affecting sperm quality were not examined. For instance, DNA fragmentation, oxidative stress levels and hormone balance, which are highly relevant in understanding how obesity impacts sperm function, were not assessed. Including these metrics would provide a more comprehensive understanding of how BMI impacts reproductive health. Since the study lacks longitudinal follow-up, like natural pregnancy rate, it cannot capture the progression or improvement of sperm quality over time relative to changes in BMI. A longitudinal study design would allow researchers to observe how weight loss or weight gain directly affects sperm parameters and fertility outcomes, providing stronger evidence of a causal relationship between BMI and sperm quality. Although the study discusses how obesity influences sperm quality through hormonal and metabolic pathways, direct hormonal measurements (e.g., testosterone, estradiol levels) and metabolic markers (e.g., insulin resistance) were not included. Adding these data would allow a deeper exploration of the physiological mechanisms linking obesity to reproductive health, providing a clearer picture of how adiposity affects hormonal balance and spermatogenesis.

An important limitation of this study is the reliance on body mass index (BMI) as the primary anthropometric measure of obesity. While BMI is widely used due to its simplicity and accessibility, it does not distinguish between fat mass and lean mass, nor does it account for fat distribution—particularly central (visceral) adiposity, which has been shown to have a more direct impact on metabolic health and reproductive function. Central obesity, rather than overall BMI, may be more strongly associated with disruptions in hormonal balance, testicular function, and semen quality [24].

In this context, Wang et al. demonstrated that central obesity, defined by metrics such as waist circumference, waist-to-hip ratio, and waist-to-height ratio, is significantly associated with reductions in semen volume, total sperm number, and sperm motility measures among 4513 Chinese sperm donation volunteers [25]. Specifically, it resulted in a 0.27 mL reduction in semen volume and increased the odds of semen volume being below reference values.

Finally, we must be acknowledged is the intrinsic variability and limited predictive value of basic semen analysis in assessing male reproductive potential. While semen parameters such as concentration, motility, and morphology are routinely used as surrogate markers of fertility, they do not reliably predict actual reproductive outcomes. It is well documented that some men with abnormal semen profiles may still achieve natural conception, while others with normal parameters may experience infertility. This diagnostic gap is further illustrated by the fact that approximately 15% of all infertility cases are classified as unexplained. Therefore, while our findings highlight obesity-related alterations in conventional semen metrics, these results should be interpreted with caution and within the broader context of reproductive function, which is influenced by a complex interplay of molecular, hormonal, genetic, and female partner-related factors [26, 27].

Future studies may benefit from incorporating more precise assessments of adiposity, such as waist circumference, waist-to-hip ratio, or imaging-based body composition analysis, to better elucidate the relationship between obesity and male reproductive health.

The study does not consider genetic predispositions that may influence both obesity and sperm quality. Genes involved in metabolism, endocrine function, and reproductive health can significantly contribute to individual variations in sperm quality, independently of BMI. Future research could incorporate genetic testing to assess the role of hereditary factors in obesity-related changes in sperm parameters. These limitations underscore the need for further studies that adopt a prospective, multicenter design, include a wider range of sperm and hormonal parameters, and control for confounding lifestyle factors. Addressing these gaps would enhance the understanding of the intricate relationship between BMI, obesity, and male reproductive health.

## Conclusion

Our findings suggest that BMI is associated with subtle but measurable alterations in semen parameters, though these effects are neither uniform across all semen characteristics nor consistent across BMI categories. While regression analyses demonstrate significant negative associations between BMI and certain parameters—such as sperm motility and volume in overweight men, and total sperm count and volume in obese men—other parameters, including morphology and concentration, were not consistently affected. Specifically, we observed significant yearly declines in total sperm count (TSC) and sperm morphology across different BMI categories. Normal weight patients exhibited significant reductions in sperm morphology, while overweight patients showed significant decreases in sperm motility and morphology. Obese patients experienced significant declines in TSC. These findings highlight the importance of maintaining a healthy weight to preserve male reproductive health, emphasizing the need for targeted interventions to address obesity-related fertility issues.

These mixed results underscore the complexity of the relationship between adiposity and male reproductive health and suggest that BMI alone may not fully capture the multifactorial pathways influencing semen quality. 

## Data Availability

Data are available upon request.

## Abbreviations

BMI: Body Mass Index
WHO: World Health Organization
TSC: Total Sperm Count
A + B Motility: Rapid and progressive sperm motility categories A and B
r: Correlation coefficient
p: p-value (statistical significance)
AB Motility: Combined sperm motility categories A (rapid) and B (progressive)
